# K-Homology Splicing Regulatory Protein (KSRP) Augments Survival and Proliferation of Human Melanoma Cells

**DOI:** 10.3390/cimb47050356

**Published:** 2025-05-13

**Authors:** Harunur Rashid, Mohammad Asif Sherwani, Jung Vin Seo, Azeem Ahmad, Sumaiya Tasnim, Quamarul Hassan, Nabiha Yusuf

**Affiliations:** 1 Department of Dermatology, Heersink School of Medicine, University of Alabama at Birmingham, Birmingham, AL 35294, USA; 2 College of Arts and Sciences, University of Alabama at Birmingham, Birmingham, AL 35294, USA; 3 Department of Oral and Maxillofacial Surgery, School of Dentistry, University of Alabama at Birmingham, Birmingham, AL 35294, USA

**Keywords:** KSRP, RNA-binding protein, mRNA-decay, melanoma

## Abstract

Melanoma is one of the most aggressive and fatal cancers; however, effective and long-lasting treatment options for melanoma continue to be sought after due to the development of resistance mechanisms to the currently available therapies. Background: The K-homology-type splicing regulatory protein (KSRP) is an RNA-binding regulatory protein that binds to the AU-rich elements at the 3′-UTR of target mRNAs. Prior studies have demonstrated that KSRP plays a crucial role in the post-transcriptional regulation of gene expression in human melanoma. Subsequently, in this study, we further examined the role of KSRP in cell migration, colony formation, apoptosis, and tumorigenicity of human melanoma. Methods: KSRP was knocked down in two different human melanoma cell lines: A375 and SK-MEL-28, using lenti-shRNA techniques. By doing so, we studied the effects of KSRP inhibition on cell migration, colony formation, proliferation, apoptosis, and tumorigenicity in these melanoma cell lines. Results: We observed a significant decrease in cell migration, colony formation, proliferation, and tumorigenicity, while also observing a substantial increase in apoptosis in the KSRP knock down melanoma cell lines. Conclusions: Our data establishes that KSRP plays a vital role in cell migration, colony formation, proliferation, apoptosis, and tumorigenicity in both the A375 and SK-MEL-28 human melanoma cell lines.

## 1. Introduction

Melanoma is one of the most aggressive and lethal forms of cancer, accounting for only about 1% of skin cancers, yet remaining the primary cause for the majority of skin cancer-related deaths [1]. Currently, in the United States, melanoma is projected to account for approximately 80% of deaths related to skin cancer and is estimated to be the fifth most common cancer in both men and women [1]. Although significant advancements in melanoma treatments have been made, mainly in immunotherapies and targeted therapies, subsequent resistance and the development of resistance mechanisms to these modalities through the activation of additional survival pathways continue to limit the long-term efficacy of these available treatment options [2,3]. Consequently, the identification and detailed study of other regulators of melanoma growth remain necessary to further develop more lasting and successful treatment options for advanced melanoma. Therefore, as previously introduced and discussed in prior research, K-homology-type splicing regulatory protein (KSRP or KHSRP) continues to remain an attractive area of study as a potential therapeutic target for melanoma due to its role in tumor cell growth and migration [4,5,6,7,8,9].

KSRP is a multifunctional, single-stranded, nucleic acid-binding regulatory protein, primarily involved in RNA metabolism through the post-transcriptional regulation of gene expression, including alternative splicing, mRNA decay, translation, and pri-miRNA processing [3,10,11,12,13,14]. Located within the middle region of the protein, four K-homology (KH) domains interact with short, four-to-six nucleic acid sequences to allow KSRP to bind to single-stranded nucleic acids through the GXXG recognition motifs [14,15,16]. KH1 has been shown to identify G-rich regions and contain the nuclear localization sequence, while KH2 has been shown to recognize and select for A/U nucleic acid sequences [14,17,18]. Furthermore, both KH3 and KH4 have been shown to interact with G-rich regions and A/U sequences, with KH3 demonstrating a stronger preference to bind to G-rich regions [14,17,18]. Nevertheless, cooperation between at least two of these KH domains is required for functional binding and stronger interactions with single-stranded nucleic acids [14,17,18]. Finally, containing a nuclear localization signal and a PG-rich region in its N-terminus, as well as a repetitive Q-rich region in its C-terminus, the primary structure of KSRP indicates the extent to which KSRP is involved in numerous molecular processes located in different regions throughout the cell [14,17,18].

In the nucleus, KSRP primarily binds to the G-rich sequences at the terminal loop of miRNA strands to assist the Drosha complex in mediating miRNA biogenesis [14,19]. Within the nucleus, KSRP also functions as a transcription factor for c-myc and interacts with complexes involved in mRNA maturation [14]. While, in the cytoplasm, KSRP preferentially binds to the AU-rich elements (AREs) at the 3′-untranslated regions (3′-UTR) of target mRNAs to regulate mRNA stability [14,20,21]. KSRP also assists the Dicer complex to help complete the maturation process for a subset of miRNAs [12,14,22]. However, there are several factors to consider that modulate KSRP function. Phosphorylation of KSRP by p38 results in an inactive cytoplasmic state to protect A/U-rich myogenic transcripts and up-regulate proinflammatory cytokines [14,23]. Additionally, changes in the signaling pathway that promote PI3K/AKT result in the transfer of KSRP into the nucleus to regulate miRNA biogenesis inhibiting proinflammatory mediators in macrophages [14,24,25].

With its association with multiple molecular processes, KSRP has been shown to be involved in the development, growth, and/or metastasis of several types of cancer, such as hepatocellular carcinoma, osteosarcoma, leukemia, lymphoma, colorectal cancer, hematologic malignancies, breast cancer, human lung cancer, and melanoma [3,5,12,14,26,27,28,29]. When examined specifically within the context of melanoma, a recent study using patient survival information has revealed that KSRP expression is up-regulated in both primary and metastatic human melanoma, with a positive correlation between higher KSRP expression and poor survival outcomes in patients [3,14]. Furthermore, KSRP has been demonstrated to play a substantial role in melanoma cell growth and proliferation. Knocking down KSRP expression in several melanoma cell lines—B16-F10, A375, and SK-MEL-28—has been shown to reduce cell proliferation, while the overexpression of KSRP in the melanoma cell line SK-MEL-28 has been shown to promote cell proliferation [3]. One such mechanism that has been identified through which KSRP modulates melanoma cell growth is the regulation of KLLN expression by RNA–protein interaction [3,14]. KLLN has been identified as a p53-regulated nuclear inhibitor of DNA synthesis and is critical for p53-induced apoptosis [3,30]. By binding to the ARE in the 3′-UTR of KLLN mRNA, KSRP promotes KLLN mRNA decay, and this interaction has been positively correlated with cell growth and proliferation in melanoma [3]. Knock down (KD) of KSRP has been shown to increase KLLN expression and inhibit melanoma cell growth, while the overexpression of KSRP has demonstrated the opposite effect and decreased KLLN expression, restoring melanoma cell growth [3]. Thus, with more insight into the effect of KSRP on gene expression and melanoma growth, this topic continues to be a promising subject of research to better understand and explore.

Accordingly, in this study, we further examined the role of KSRP in two different melanoma cells lines: A375 and SK-MEL-28. Using lenti-shRNA techniques, we knocked down KSRP in A375 and SK-MEL-28 to investigate the role of KSRP in melanoma cell migration, colony formation, proliferation, apoptosis, and tumorigenicity. In KSRP knock down (KD) melanoma cells, colony formation, proliferation, migration, and wound healing were observed to decrease significantly. Additionally, we assessed the effect of KSRP KD in the development of melanoma by injecting A375 and SK-MEL-28 into athymic nude mice and noted consistent observations, primarily a substantial reduction in tumor weight and volume in mice with A375 or SK-MEL-28 KSRP KD compared to those of the control. Ultimately, these findings indicate that KSRP is an essential regulatory factor for the cell migration, colony formation, proliferation, and apoptosis of both the A375 and SK-MEL-28 melanoma cell lines, strongly suggesting the significant potential of KSRP as a lasting effective potential therapeutic target for advanced melanoma.

## 2. Materials and Methods

### 2.1. Cell Culture and Transfection of Human Melanoma Cells

Human melanoma cell lines A375 and SK-MEL-28 and 293 T cells were purchased from American Type Culture Collection (ATCC, Rockville, MD, USA). Cells were seeded in Dulbecco’s Modified Eagle’s Medium (DMEM, Thermo Fisher Scientific, Waltham, MA, USA) with 10% fetal bovine serum (Thermo Fisher Scientific, Waltham, MA, USA), 100 U/mL penicillin, and 100 μg/mL streptomycin (Thermo Fisher Scientific, Waltham, MA, USA) at 37 °C in 5% CO_2_. For KSRP knoc down, 293 T cells (5 × 10^6^/well) were transfected with KSRP shRNA (Dharmacon A Horizon Discovery Group company, Lafayette, CO, USA) or empty vector (pLVX-IRES-Puro, 50 nM, (Addgene, Watertown, MA, USA), using PolyJet reagent (SignaGen Laboratories, Rockville, MD, USA) according to the manufacturer’s protocol. After transfection, cells were incubated at 37 °C for 48 h. Subsequently, the 293T lentiviral supernatant, containing the retroviral particles, was harvested using centrifugation (956 g, 15 min). For transduction, supernatant was passed through a 45 μm filter (Costar, Cambridge, MA, USA) to obtain viral particles, which were then added to A375 or SK-MEL-28 cells (5 × 10^6^/well). After 48 h of transduction, cells were selected with puromycin (Sigma, St. Louis, MA, USA).

### 2.2. Preparation of Cell Lysates and Western Blot Analysis

For the Western blot analysis of KSRP, lysates were prepared using 0.05 mL of RIPA buffer containing 20 mM HEPES, pH 7.4, 2 mM EDTA, 250 mM NaCl, 0.1% NP-40, 2 mg/mL leupeptin, 2 mg/mL aprotinin, 1 mM PMSF, 0.5 mg/mL benzamidine, 1 mM dithiothreitol, and 1 mM sodium vanadate. About 30–40 µg protein was loaded in each well, resolved on 12% SDS-polyacrylamide gel, and transferred onto PVDF membranes. Membranes were incubated in a blocking buffer (5% non-fat milk) for 1 h and then incubated with the primary antibody (Anti-KSRP, clone Ab5 Cat# MABE987 Millipore) in a blocking buffer (0.5 μg/mL) overnight at 4 °C. The membrane was then washed with TBS-T and incubated with a secondary antibody conjugated with horseradish peroxidase (Anti mouse IgG HRP-linked (1:3000) Cat# 7076P2, Cell Signaling Technology, Danvers, MA, USA). Protein bands were visualized using the enhanced chemiluminescence detection system (iBright Western Blot imaging systems, Thermofisher Scientific, Waltham, MA, USA). To verify the equal protein loading and the transfer of proteins from gel to membrane, the blots were probed for GAPDH (1:1000), (GAPDH Loading Control Monoclonal Antibody Cat#MA5-15738, Themofisher Scientific, Waltham, MA, USA).

### 2.3. Clonogenic Assay

KSRP KD and wild-type (WT) A375 and SK-MEL-28 cells (200–300 cells per well) were seeded in 6-well plates. After incubation for 2 weeks, colonies were stained with crystal violet and the number of colonies was determined as describer earlier [31].

### 2.4. Cell Migration Assay

A wound healing assay was performed to examine cell migration. KSRP KD and wild-type (WT) A375 and SK-MEL-28 cells were seeded in 12-well cell culture plates and incubated overnight. Using a sterile 200 μL pipette tip, the monolayer of cells was scratched. Cells were washed with PBS and then incubated in complete medium. Cell growth and migration were determined by imaging the scratch using a phase-contrast microscope every 24 h for the next 3 days [32].

### 2.5. Apoptosis Assay

The FITC Annexin V method was used to quantitatively determine the percentage of apoptotic cells as described earlier [31]. KSRP KD and wild-type (WT) A375 and SK-MEL-28 cells (10^3^) were seeded in 12-well cell culture plates and incubated overnight. Cells were trypsinized and washed twice with cold PBS and then suspended in 1× Binding Buffer at a concentration of 1 × 10^6^ cells/mL. A one-hundred-microliter cell solution (1 × 10^5^ cells) was transferred in a 5 mL culture tube. Five microliters of FITC Annexin V and 10 µL of PI were added and incubated for 15 min at RT (25 °C) in the dark. Binding Buffer (400 µL) was added, and cells were analyzed using a flow cytometer.

### 2.6. Tumor Growth in Nude Mice

KSRP KD and wild-type (WT) A375 and SK-MEL-28 cells (10^6^) were injected subcutaneously into the right and left flanks, respectively, of athymic nude mice (Jacksons Laboratories, Bar Harbor, ME, USA). Mice were monitored every alternate day for tumor volume. Tumor weight was measured at the end of experiment. Mice were sacrificed at the end of experiment, and tumors were collected for analysis of biomarkers. The procedures were approved by the Institutional Animal Care and Use Committee of the University of Alabama at Birmingham (Protocol# 22587).

### 2.7. RNA Extraction and Quantitative Real-Time PCR

Total RNA from the WT and mutant A375 cell line was extracted using Trizol reagent (Life Technologies, Carlsbad, CA, USA) according to the manufacturer’s protocol. cDNA was synthesized from 1 µg RNA using an iScript cDNA synthesis kit (Bio-Rad, Hercules, CA, USA) according to the manufacturer’s instructions. Using the iQ^TM^ SYBR Green Master Mix (Bio-Rad, Hercules, CA, USA), cDNA was amplified using real-time PCR with a Bio-Rad MyiQ thermocycler and an SYBR Green detection system (Bio-Rad, Hercules, CA, USA). The standard PCR conditions were 95 °C for 10 min and then 40 cycles at 95 °C for 30 s, 60 °C for 30 s, and 72 °C for 30 s. The expression of *Caspase-1*, *Tp53INP1*, *GDF15*, *CREB3L1*, *ATF3*, *BAX*, *Bap*, and *Pten* gene (Table 1) was normalized to the expression level of the GAPDH mRNA in each sample. For mRNA analysis, the calculations for determining the relative level of gene expression were made using the cycle threshold (*C*_t_) method. The mean *C*_t_ values from duplicate measurements were used to calculate the expression of the target gene, with normalization to a housekeeping gene used as the internal control, using the formulae 2^−ΔΔCT^.

### 2.8. Statistical Analysis

All data are shown as mean *±* SD. Levels of significance of differences among the groups were calculated using unpaired two-tailed *t*-test for all the data. *p* < 0.05 was considered to be statistically significant.

## 3. Results

### 3.1. KSRP Silencing Inhibits Colony Formation and Proliferation of Melanoma Cells

**Figure 1:**
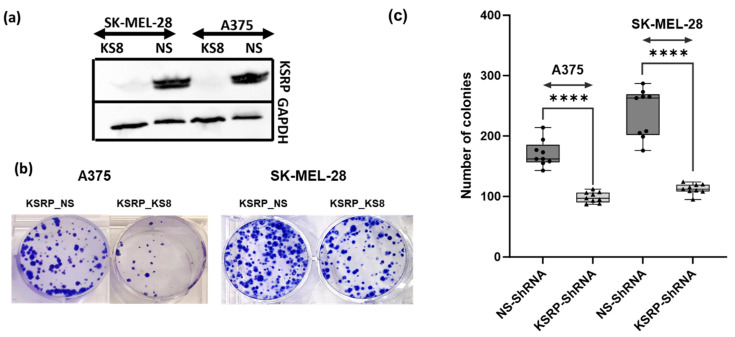
Silencing KSRP decreases melanoma colony formation and proliferation as observed through clonogenic assays when compared to that of the wild type. Clonogenic assays of A375 and SK-MEL-28 cells transfected with shNS or shKSRP. (**a**) Western blot to show shKSRP knock down in A375 and SK-Mel-28 melanoma cell lines (**b**) Top panel shows a macroscopic view of the colonies stained with 0.1% crystal violet. After 2 weeks, the number of colonies was (**c**) counted and graphed. A representative picture from three independent experiments is shown here. All data are mean ± SD. **** *p* < 0.0001.

To determine the role of KSRP in melanoma colony formation and proliferation, we knocked down the expression of KSRP using lenti-shRNA techniques in A375 and SK-MEL-28 cells. To validate the knock down of KSRP, Western blot analysis was performed on cell lysates using the method discussed in Section 2. A complete reduction in KSRP levels was observed in the knock down samples compared to controls, indicating successful silencing of KSRP. GAPDH was used as a loading control to confirm equal protein loading and transfer efficiency across all samples. Knock down of KSRP reduced colony formation and proliferation determined using clonogenic assay. We observed significant reductions in the number of colonies formed when KSRP was knocked down when compared to the number of colonies formed by WT cells (Figure 1). These observations suggest that silencing KSRP inhibits colony formation and proliferation of A375 and SK-MEL-28 melanoma cells.

### 3.2. KSRP Silencing Inhibits Migration of Melanoma Cells

**Figure 2:**
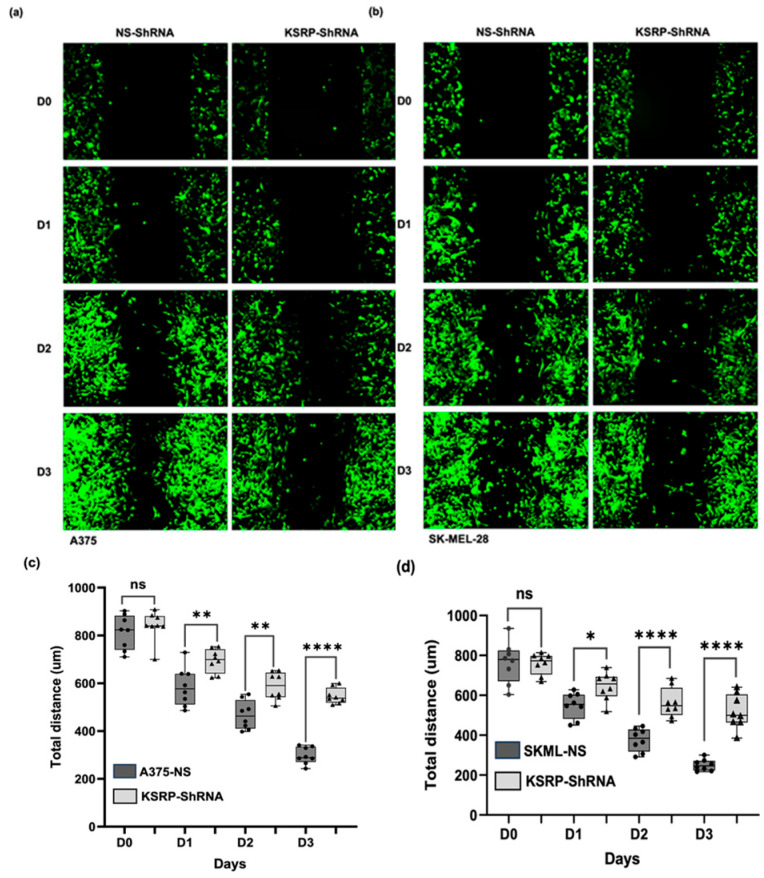
Silencing KSRP decreases melanoma cell migration and wound healing capacity as observed through cell migration assays when compared to that of the wild type. (**a**,**b**) Macroscopic view of the cell migration assays of (**a**) A375 and (**b**) SK-MEL-28 cells transfected with shNS or shKSRP during 3 consecutive days. (**c**,**d**) The total distance of the scratch was measured and graphed over 3 consecutive days starting on the day the wound was made (D0) of the (**c**) A375 and (**d**) SK-MEL-28 melanoma cell lines. A representative picture from three independent experiments is shown here. All data are mean ± SD. * *p* < 0.05, ** *p* < 0.01, **** *p* < 0.0001, ns = not significant.

To study the role of KSRP in cell migration, we performed wound healing assays. Briefly, we knocked down the expression of KSRP using lenti-shRNA techniques in A375 and SK-MEL-28 cells. We then performed cell migration assays of both the wild-type and knock down cell lines to examine differences in cell migration and wound healing. Knock down of KSRP significantly inhibited cell migration and wound healing capacity in A375 and SK-MEL-28 melanoma cell lines (Figure 2). These results suggest that the inhibition of KSRP expression reduces the migration capacity of A375 and SK-MEL-28 cells.

### 3.3. KSRP Silencing Increases Apoptosis of Melanoma Cells

**Figure 3:**
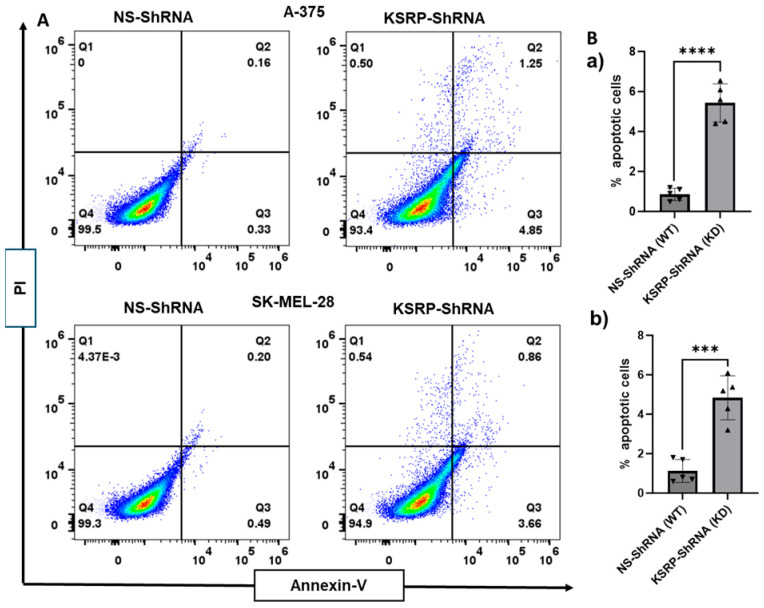
Silencing KSRP induces apoptosis in melanoma cells as observed through FITC Annexin V assay when compared to the wild type. (**A**) Flow cytometry plot of cells stained using the Annexin V FITC/PI kit, which distinguishes apoptotic cells (Annexin V positive, PI positive, Annexin V positive and PI negative) from necrotic cells (PI positive and Annexin V negative) and viable cells (Annexin V negative, and PI negative) (**a**) A375 and (**b**) SK-MEL-28 cells transfected with shNS or shKSRP. (**B**) Quantification of apoptosis results. A representative picture from three independent experiments is shown here. All data are mean ± SD. *** *p* < 0.001, and **** *p* < 0.0001.

KSRP KD and wild-type (WT) A375 and SK-MEL-28 cells were studied for apoptosis using the FITC Annexin V assay as described in Section 2. The assay was performed to quantitatively determine the percentages of cells within the knock down population that are undergoing apoptosis. Knock down of KSRP significantly increases the percentages of apoptosis within the population of A375 and SK-MEL-28 melanoma cell lines (Figure 3). 

### 3.4. KSRP Silencing Inhibits Melanoma Tumor Growth in Nude Mice

**Figure 4:**
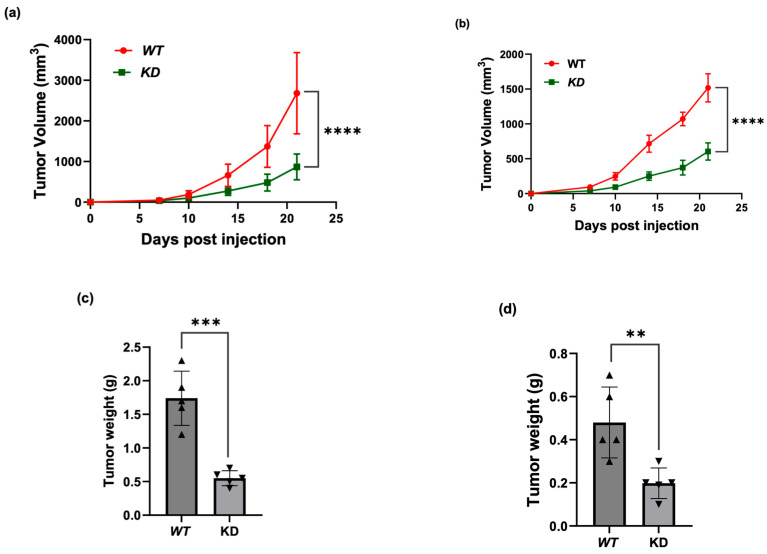
Knock down KSRP decreases the tumorgenicity of melanoma cell lines as observed in vivo compared to that of the wild type. (**a**,**b**) Tumor volume decreased in mice injected with KSRP knocked down melanoma cell lines (**a**) A375 and (**b**) SK-MEL-28 compared to those of the wild type. (**c**,**d**) Tumor weight also decreased in mice injected with KSRP knocked down melanoma cell lines (**c**) A375 and (**b**) SK-MEL-28 when compared to those of wildtype. Experiments were conducted in 6 mice per group and each experiment was repeated twice. All data are mean ± SD. ** *p* < 0.01, *** *p* < 0.001, and **** *p* < 0.0001.

To understand the role of KSRP in the tumorgenicity of melanoma, we knocked down the expression of KSRP using lenti-shRNA techniques in A375 and SK-MEL-28 cells. We then subcutaneously injected the WT and KD melanoma cells into the left and right flanks, respectively, of nude mice to study the effects in vivo. We observed a significant reduction in tumor volume in mutant mice compared to those of the WT three weeks post injection (Figure 4). These observations suggest that silencing KSRP expression reduced tumorgenicity in melanoma cell lines.

### 3.5. KSRP Silencing Alters Gene Expression

**Figure 5:**
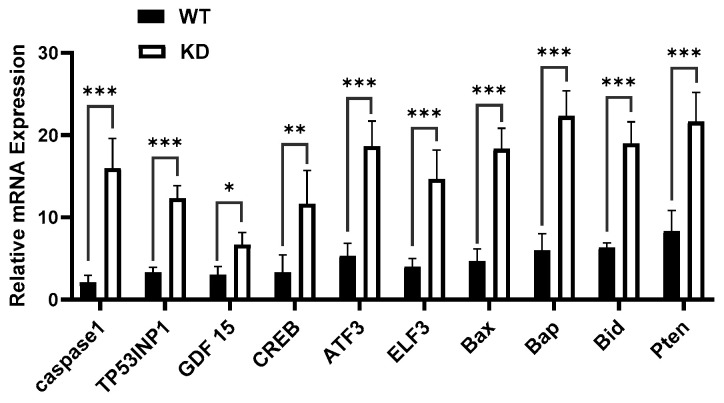
Manipulation of KSRP expression alters gene expression. Expression of 10 genes, Caspase1, TP53INP1, GDF15, CREB, ATF3, ELF3, Bax, Bap, Bid, and Pten, was up-regulated in transfected cells analyzed using qRT-PCR. A representative picture from three independent experiments is shown here. All data are mean ± SD. * *p* < 0.05, ** *p* < 0.01, and *** *p* < 0.001.

To elucidate the mechanism through which KSRP regulates melanoma cell growth, we knocked down the expression of KSRP using lenti-shRNA techniques in the A375 melanoma cell line. We then performed qRT-PCR samples isolated from the KSRP knock down A375 cells and the wild type to identify genes and/or transcripts with altered expression. We found that 10 genes/transcripts were up-regulated upon KSRP silencing (Figure 5). These results provide additional areas of study to further investigate the role of KSRP on melanoma cell growth through these cellular mechanisms.

## 4. Discussion

In this study, we demonstrate that KSRP plays a critical role in melanoma cell migration, colony formation, and proliferation by repressing the expression of KSRP using lenti-shRNA techniques in A375 and SK-MEL-28 human melanoma cells in vitro. We also examined the role of KSRP in apoptosis in the mutated and wild-type human melanoma cell lines. We observed a positive correlation between KSRP expression and colony formation, proliferation, and migration, and a negative correlation between KSRP expression and apoptosis. Silencing KSRP appeared to significantly reduce cell migration, colony formation, and proliferation, while increasing the percentage of apoptosis of the A375 and SK-MEL-28 melanoma cell lines. These observations suggest that in human melanoma cell lines, KSRP is significantly important for melanoma progression, further supporting and supplementing the existing literature on KSRP and its role in cancer.

As introduced earlier, previously conducted studies have revealed that KSRP plays various roles in both advancing and inhibiting the development and growth of many types of human cancer. KSRP has been shown to promote the progression and proliferation of hepatocellular carcinoma, osteosarcoma, leukemia, lymphoma, colon cancer, and hematologic malignancies [5,12,14,26,27]. Similarly, KSRP has also been shown to promote metastasis in breast cancer, human lung cancer, and melanoma [3,14,28,29]. However, in contrast, in non-small-cell lung cancer and glioblastoma cells, KSRP has been reported to inhibit migration, cell invasion, and metastasis accordingly [3,8,29]. Consequently, further study to better understand the roles of KSRP and its associated cellular mechanisms in different human cancers remains essential if KSRP is to be targeted as a possible future therapeutic in cancer therapies and treatments.

Several mechanisms through which KSRP is involved in different types of cancer have been identified and assessed so far. KSRP has been studied to suppress cell invasion and metastasis in non-small-cell lung cancer by regulating EGR3 mRNA degradation through miR-23a [3,29]. In contrast, higher levels of KSRP expression increase the maturation of cancer-associated miRNAs, resulting in the enhanced growth, migration, and invasion of esophageal squamous cell carcinoma cells [14,41]. Similarly, increased levels of KSRP expression in BRCA1-mutated breast and ovarian cancers were observed through the interaction between KSRP and lncRNA LINC01305 [14,42]. Over-expression of KSRP in hepatocellular carcinoma has also been shown to positively correlate with c-myc transcription and, therefore, increase the progression of hepatocellular carcinoma [14,27]. Additionally, through its interactions with the small nucleolar RNAs SNORA18 and SNORA22, KSRP has been demonstrated to promote the invasiveness and metastasis of pancreatic cancer cells [14,43]. In colorectal cancer, inhibiting KSRP using the DKC1125 protein has been shown to limit metastasis through the degradation of KITENIN, suggesting that KSRP plays a significant role in promoting the progression of colorectal cancer [14,44]. And finally, in melanoma, by binding to the ARE in the 3′-UTR of KLLN mRNA, KSRP has been observed to promote KLLN mRNA decay, resulting in increased cell growth and proliferation [3]. Thus, it is likely that KLLN plays a role in promoting the significant reduction in melanoma cell migration, colony formation, and proliferation, while also contributing to the increase in apoptosis that we observed in this study.

In order to identify several possible factors involved in the decrease in melanoma cell growth that we observed after silencing KSRP expression, we were able to identify 10 up-regulated genes/transcripts (Figure 5) in the KSRP-deficient samples, suggesting that there may be other cellular processes contributing to the regulation of melanoma growth and proliferation. The up-regulation of caspase-1 implicates the induction of pyroptosis and a reduction in cell proliferation and migration, consistent with our observations [45]. Similarly, the up-regulation of Tp53INP1 indicates the activation of autophagy-dependent cell death, limiting cell growth and proliferation [46]. High levels of ATF3 expression have also been shown to inhibit melanoma cell growth, cell migration, and tumor development, further supporting our findings [47]. Likewise, the up-regulation of Bid likely promotes the up-regulation and activation of BAX that we observed to stimulate the proapoptotic pathways, as demonstrated in prior studies [48,49,50,51,52,53]. Furthermore, previously conducted studies have also shown that losses and/or mutations in Bap are associated with increased susceptibility to uveal melanoma and cutaneous melanocytic lesions [54]. Thus, with its role as a tumor suppressor, the up-regulation of Bap also likely played a significant role in reducing the cell growth and proliferation observed in our studies [54]. However, higher levels of CREB3L1 expression in skin cutaneous melanoma have been positively correlated with poorer overall survival, suggesting that, in melanoma, CREB3L1 promotes tumor growth and development [55]. Additionally, the up-regulation of GDF15 has also been associated with advanced melanoma tumor invasion and metastasis, contradicting our findings [56,57]. Perhaps, the greater up-regulation of PTEN counters the effects of GDF15 up-regulation, as activation of PTEN expression has been implicated to reverse the influence of GDF15 on melanoma and promote host immune responses through the negative regulation of the expression of immunosuppressive cytokines, such as IL-6, IL-10, and VEGF [56,58]. Interestingly, although the role of ELF3 in melanoma has not yet been examined, previous studies have reported observing a significant downregulation of ELF3 in melanoma tumors, suggesting that expression of ELF3 may aid the regulation of melanoma growth and development [59]. Ultimately, while all of these genes may not be involved in the direct regulation of melanoma growth and development, it would be useful to further examine their roles in relation to KSRP in future studies to better understand the effects of targeting KSRP in melanoma therapies.

Our results suggest that silencing KSRP, through its subsequent post-transcriptional regulatory effects, is involved in the reduction in cell migration, colony formation, proliferation, and the increase in apoptosis of the A375 and SK-MEL-28 human melanoma cell lines. These results remain relatively consistent with the findings from the existing literature on the effects of KSRP on melanoma. Nevertheless, the approach used in this study did not explicitly examine the role of KSRP on its target proteins and signaling pathways when interrogating the effects of KSRP on cell migration, colony formation, proliferation, and apoptosis of melanoma cells. Thus, other approaches to further study these mechanisms and targets upon KSRP silencing should better elucidate these factors and their roles in the migration, colony formation, proliferation, and apoptosis of melanoma cells. In summary, this study strongly implies that targeting KSRP and its associated cellular mechanisms could serve as effective potential therapeutics for the treatment of human melanomas.

## 5. Conclusions

Ultimately, in this study, we found that KSRP plays a significant role in the cell migration, colony formation, proliferation, and apoptosis of human melanoma cell lines. By silencing KSRP expression in the A375 and SK-MEL-28 human melanoma cell lines, we were able to observe the effects of KSRP inhibition and its following post-transcriptional effects on melanoma in vitro and in vivo using a tumor xenograft mouse model. Additionally, we briefly examined the effects of KSRP inhibition on the expression of several different genes that are known targets of KSRP and found 10 genes/transcripts to be up-regulated. Both the cell lines tested have BRAF mutations. It would be interesting to see if N-ras mutant melanoma lines have a similar effect. Future studies could be conducted to better understand the underlying mechanisms by which KSRP impacts the expression of the 10 up-regulated genes/transcripts and their subsequent effects on human melanoma. Thus, from our findings, KSRP remains an attractive target and area of research for human melanoma treatment that is more effective and long-lasting.

## Figures and Tables

**Table 1:** Primer sequences used in reverse transcriptase polymerase chain reaction.

Gene	Primer Sequence	References
*GAPDH*	5′-CGACCACTTTGTCAAGCTCA-3′ 5′-AGGGGTCTACATGGCAACTG-3′	[33]
*Caspase1*	5′-CTTGGAGACATCCCACAATG-3′ 5′-CTGCCCACAGACATTCATAC-3′	[3]
*Tp53INP1*	5′-TCAGCAGAAGAAGAAGAAGAAGAG-3′ 5′-AGCAGGAATCACTTGTATCAGC-3′	[33]
*GDF15*	5′-TCACGCCAGAAGTGCGGCTG-3′ 5′-CGTCCCACGACCTTGACGCC-3′	[33]
*CREB3L1*	5′-GGAGAATGCCAACAGGACC-3′ 5′-GCACCAGAACAAAGCACAAG-3′	[34]
*ATF3*	5′-AGTCAGTTACCGTCAACAACAGA-3′ 5′-CTCAGCATTCACACTCTCCAGTT-3′	[35]
*ELF3*	5′-CCACCACCATCTTTCCGAGT-3′ 5′-TACGGGTGACAGGCTACAAA-3′	[36]
*BAX*	5′-TTTGCTTCAGGGTTTCATCC-3′ 5′-CAGTTGAAGTTGCCGTCAGA-3′	[37]
*Bap1*	5′-AGGAGCTGCTGGCACTGCTGA-3′ 5′-TTGTGGAGCCGGCCGATGCT-3′	[38]
*Bid*	5′-ATGGACTGTGAGGTCAACAACGG-3′ 5′-CACGTAGGTGCGTAGGTTCTGGTTA-3′	[39]
*PTEN*	5′-AGTTTGTGGTCTGCCAGCTA-3′ 5′-TCAGAGTCAGTGGTGTCAGA-3′	[40]

## Data Availability

Data are available upon request.
